# Two-port versus three-port video-assisted thoracoscopic surgery for primary spontaneous pneumothorax: feasibility, postoperative outcome and long-term recurrence rates

**DOI:** 10.1186/s12893-021-01426-6

**Published:** 2021-12-18

**Authors:** Stephen Fung, Hany Ashmawy, Sami Safi, Anja Schauer, Alexander Rehders, Levent Dizdar, Georg Fluegen, Wolfram Trudo Knoefel

**Affiliations:** Department of Surgery, University Hospital Duesseldorf and Heinrich-Heine-University Duesseldorf, Moorenstrasse 5, 40225 Duesseldorf, Germany

**Keywords:** Two-port VATS, Three-port VATS, Outcome, PSP

## Abstract

**Background:**

Two-port VATS (2-P-VATS) and three-port VATS (3-P-VATS) are well-established techniques for surgical therapy of primary spontaneous pneumothorax (PSP). However, comparisons of both techniques in terms of postoperative outcome and recurrence are limited.

**Methods:**

From January 2010 to March 2020, we retrospectively reviewed data of 58 PSP patients who underwent VATS in our institution. For statistical analysis, categorical and continuous variables were compared by chi-square test or Fisher’s exact test and the Student´s t-test, respectively. Twenty-eight patients underwent 2-P-VATS and 30 were treated with 3-P-VATS. Operation time, length of hospital stay (LOS), total dose of analgesics per stay (opioids and non-opioids), duration of chest tube drainage, pleurectomy volume (PV), postoperative complications and recurrence rates were compared between both groups**.**

**Results:**

Clinical and surgical characteristics including mean age, gender, Body-Mass-Index (BMI), pneumothorax size, smoking behaviour, history of contralateral pneumothorax, side of pneumothorax, pleurectomy volume and number of resected segments were similar in both groups. The mean operation time, LOS and total postoperative opioid and non-opioid dose was significantly higher in the 3-P-VATS group compared with the 2-P-VATS group. Despite not being statistically significant, duration of chest tube was longer in the 3-P-VATS group compared with the 2-P-VATS group. In terms of postoperative complications, the occurrence of hemothorax was significantly higher in the 3-P-VATS group (3-P-VATS vs. 2-P-VATS; p = 0.001). During a median follow-up period of 61.6 months, there was no significant statistical difference in recurrence rates in both groups (2/28 (16.7%) vs. 5/30 (7.1%); p = 0.274).

**Conclusion:**

Our data demonstrate that 2-P-VATS is safer and effective. It is associated with reduced length of hospital stay and decreased postoperative pain resulting in less analgesic use.

## Background

As defined in the current German S3 guidelines, primary spontaneous pneumothorax (PSP) describes the presence of air without preceding trauma or underlying pulmonary disease within the pleural space of young patients under 45 years of age [1] The incidence of PSP has been reported with approximately 1–9.8 and 7–24 cases per 100,000 individuals per year in females and males respectively [2, 3] Due to the low recurrence und morbidity rates, current guidelines [1, 4, 5] recommend VATS for surgical treatment of PSP. In the last decades, thoracic surgery has evolved from thoracotomy to video-assisted thoracoscopic surgery (VATS). While three-port VATS (3-P-VATS) remains the gold standard for PSP treatment, recent publications have demonstrated the advantages of uniportal VATS in terms of postoperative pain and paraesthesia, analgesic use, length of hospital stay, cosmetic results and patient satisfaction scores in specialized settings [6–11]. However, only a few studies have analysed and compared the postoperative outcome of the widely implemented two-port VATS (2-P-VATS) with 3-P-VATS [8, 12]. Therefore, the aim of this study was to evaluate and compare the outcome and long-term recurrence rates of PSP patients treated with 2-P-VATS and 3-P-VATS in our institution.

## Material and methods

### Patients

We retrospectively analysed data of 58 patients who underwent video-assisted thoracoscopic surgery (VATS) for primary spontaneous pneumothorax (PSP) in our institution between January 2010 and March 2020. Twenty-eight patients were treated using 2-P-VATS and 30 patients underwent the conventional 3-P-VATS. Indication for surgery was persistent air leak for more than 5 days after chest tube treatment on first episode (2-P-VATS: N = 8; 3-P-VATS: N = 10), second ipsilateral pneumothorax (2-P-VATS: N = 12; 3-P-VATS: N = 15), synchronous bilateral spontaneous pneumothorax (2-P-VATS: N = 5; 3-P-VATS: N = 3), and spontaneous hemopneumothorax (2-P-VATS: N = 3; 3-P-VATS: N = 2). Prior to surgery, all the patients received a CT (computer tomography) scan of the thorax to detect any bullous disease. For each patient, medical charts were reviewed to retrieve the following variables: age, sex, body mass index (BMI), side of pneumothorax, pneumothorax size, smoking behaviour, number of resected lung segments (if any), volume of resected parietal pleura, length of hospital stay (days), duration of chest drainage (days), total dose of opioid and non-opioid use per stay, operation time and postoperative complications (Tables 1 and 2). Only PSP patients with completed follow-up data were included in this study. All the patients underwent VATS with partial pleurectomy and bullectomy when blebs where evident. Patients who underwent other treatment modalities such as thoracotomy, apical pleurectomy or suffered a different pneumothorax type (e.g. secondary spontaneous pneumothorax, catamenial pneumothorax, iatrogenic pneumothorax) were excluded from this study.

**Table 1:** Clinical characteristics

Variables	Two-port VATS N = 28	Three-port VATS N = 30	*p-*value
Age (years)	23 (range 18–40)	22 (range 18–39)	0.49
Sex (n; %)			0.97
Female	5 (17.9%)	5 (16.7)	
Male	23 (82.1%)	25 (83.3%)	
Weight (kg)	70	63	0.53
Height (m)	1.80	1.80	0.77
BMI (kg/m^2^)	21.35	20.15	0.51
Side of pneumothorax			0.54
Right	22 (78.6%)	15 (50%)	
Left	6 (21.4%)	15 (50%)	
Collins (A + B + C) (cm)	8.99	9.4	0.95
Active smoker			0.445
Yes	7 (25%)	5 (16.7)	
No	21 (75%)	25 (83.3%)	
History of pneumothorax	1	3	0.336

Unless otherwise specified, all data are presented as mean value. *BMI* Body-Mass-Index, *cm* centimetre, *Kg* kilogram, *m* metre, A *p-value < 0.05 indicates statistical significance. Collins (A + B + C) = sum of the intrapleural distances (cm) according to the regression formula derived from Collins et al. (13)

**Table 2:** Surgical characteristics

Variables	Two-port VATS N = 28	Three-port VATS N = 30	*p-*value
Chest tube duration (days)	5,5	7	0.228
LOS (days)	7	9	0.012*
Piritramide dosage / stay (mg)	15	30	0.012*
Non-opioid dosage / stay (g)	16	20	0.010*
Operation time (min)	65	90	0.001*
Length of air leak (days)	5	6	0.135
Pleurectomy volume (cm^3^)	12.8	14.3	0.450
Postoperative complications			
Hemothorax (n, %)	0	3 (10%)	0.001*
Prolonged air leak (n, %)	9 (32.1%)	15 (50.0%)	0.136
Recurrence (n, %)	2 (7.1%)	5 (16.7%)	0.274

Unless otherwise specified, all data are presented as mean value. *cm*^*3*^ cubic centimetre, *min* minutes, *mg* milligram, *g* gram, *LOS* length of hospital stay. A *p-value < 0.05 indicates statistical significance

The pneumothorax size was assessed using the regression formula derived from Collins et al*.* [13]. The volume of the resected parietal pleura was measured in cubic centimeter (cm^3^) as noted in the pathology results. The operation time (minutes) was defined as the time from incision to the end of skin closure. A postoperative prolonged air leak was defined as a persistent air leakage for more than 5 days. Recurrence was described as pneumothorax detected on a chest radiograph at presentation in our emergency room after surgical treatment by VATS or chest tube drainage. Our standard postoperative medication regime of analgesia (non-opioid) was administered intravenously or orally. The patients received either Metamizol-Natrium 1000 mg, Paracetamol 1000 mg or Ibuprofen 600 mg four times per day. In case of persistent pain using the standard pain medication regime, we applied Piritramide (opioid) 7.5 mg intravenously every 4–6 h on patient request. For each patient, the total opioid- and non-opioid dose per stay was evaluated and documented.

One week after discharge from the hospital, the patients visited our outpatient clinic for postoperative control and follow-up. These visits continued in 3 months intervals for one year. For long-term follow-up, the patients were contacted (by telephone call or mail) and assed with a questionnaire. The median follow-up period was 61.6 (range 5–119) months.

The local Institutional Review Board of the Heinrich- Heine University Hospital of Duesseldorf approved this study (study Nr: 2020-1271).

#### Surgical procedures

Excluding the amount of surgical ports, all patients received the same surgical treatment, consisting of partial pleurectomy and bullectomy when blebs were evident. All operations were performed under general anaesthesia and one lung-ventilation. The patients were placed in a lateral position and the table flexed up to 35° to open up the intercostal spaces.

#### ***Three-Port VATS (Fig. ******1******)***

**Fig. 1:**
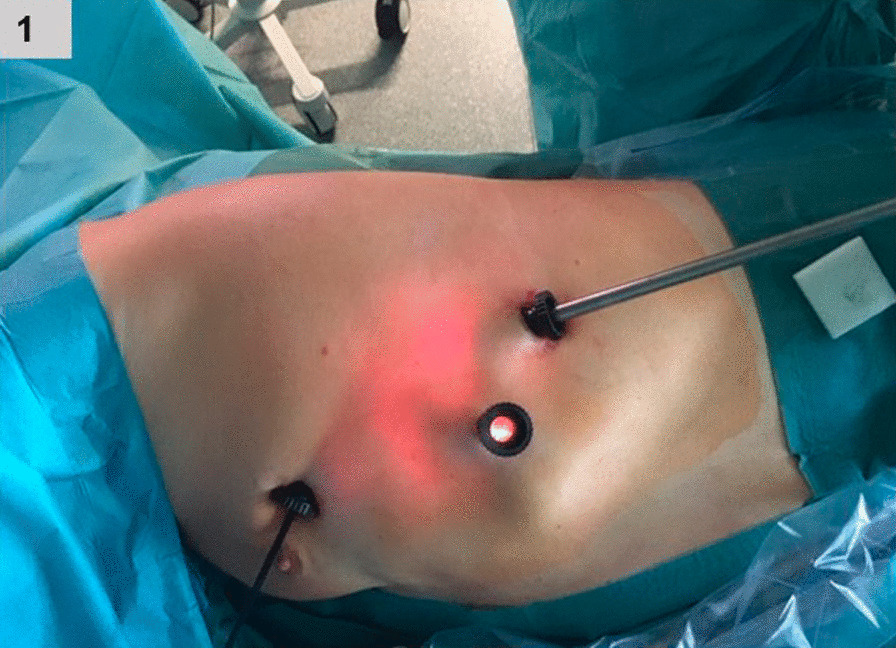
Trocar position during three-port VATS

For 3-P-VATS, the first 1.5 cm skin incision was performed at the level of the 5^th^ intercostal space in the anterior axillary line. After placement of an 11 mm trocar and insertion of the video-thoracoscope, explorative thoracoscopy was performed for thorough inspection of the visceral and parietal pleura. If the patient already had a chest tube, this chest thoracostomy wound (mostly 5th or 6th intercostal space of the mid-axillary line) was used for the 11 mm optical trocar. Under thoracoscopic control, two additional 11 mm trocars were placed at the level of the 7th and 8th intercostal space in the mid- and posterior-axillary line, respectively (Fig. 1). In case of bullae or blebs, the video-thoracoscope was removed and reinserted through the trocar in the 8th intercostal space. Hereafter, an endograsper and an endoscopic stapling device (Autosuture GIA Universal; Covidien, Mansfield, MA, USA) were inserted through the trocars in the 5th and 7th intercostal space, respectively. After grasping the bullous area, bullectomy was undertaken with the stapling device. Partial pleurectomy was performed from the apex of the pleura cavity up to the 7th or 8th intercostal space in a blunt manner using an endograsper and a blunt dissector (Endo Peanut™ Auto suture™, COVIDIEN ®, Willow Lane, Mokena, IL), while sparing the regions of the subclavian and internal mammary vessels to avoid damage of these structures.

#### ***Two-Port VATS (Fig. ******2******)***

**Fig. 2:**
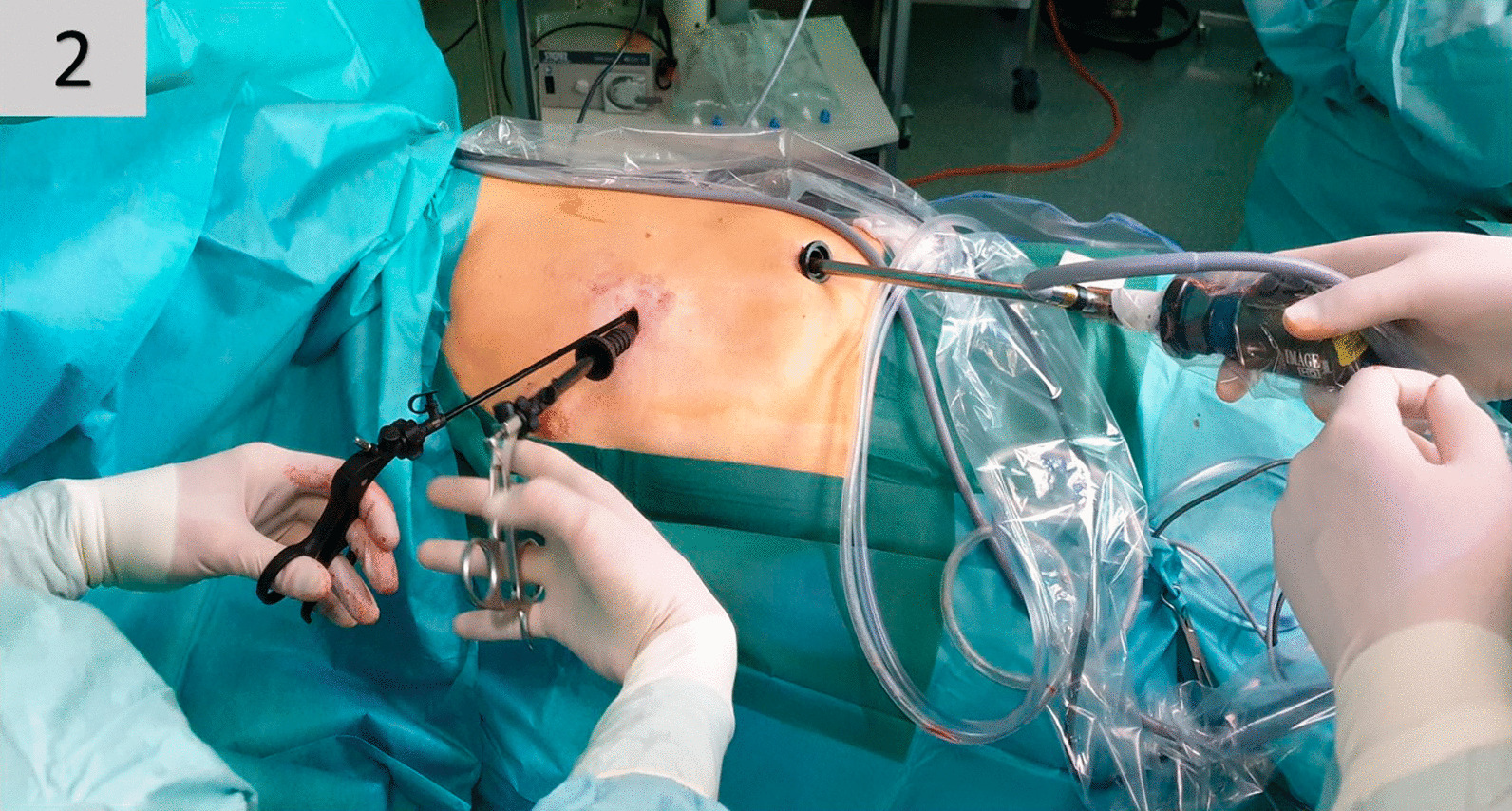
Trocar placement for two-port VATS

Two 1.5 cm skin incisions were made at the level of the 5^th^ and 8^th^ intercostal space in the mid- and anterior axillary line, respectively (Fig. 2). Thoracoscopy and pleurectomy were performed as described in 3-P-VATS. For bullectomy, an endograsper was inserted without trocar-guidance through the skin incision in the 5th intercostal space, adjacent to the trocar of endoscopic stapling device. The video-thoracoscope was introduced through the trocar in the 8th intercostal space.

In both procedures, an underwater air leak test was performed. Two 24 Fr chest tubes, placed at the apex of the thorax cavity and in the costodiaphragmatic recess, were inserted through the incisions in the 5th and 8th intercostal spaces, respectively, and connected to a digital chest drainage system (Thopaz + , Medela AG, Baar, Switzerland) with a suction equivalent to – 20 cm H_2_O.During postoperative care, the chest tube drains were removed when no clinical signs of air leak and a drain output less than 200 ml after 24 h were evident. After chest tube removal, a chest radiograph was taken to verify full expansion of the lung.

**Fig. 3:**
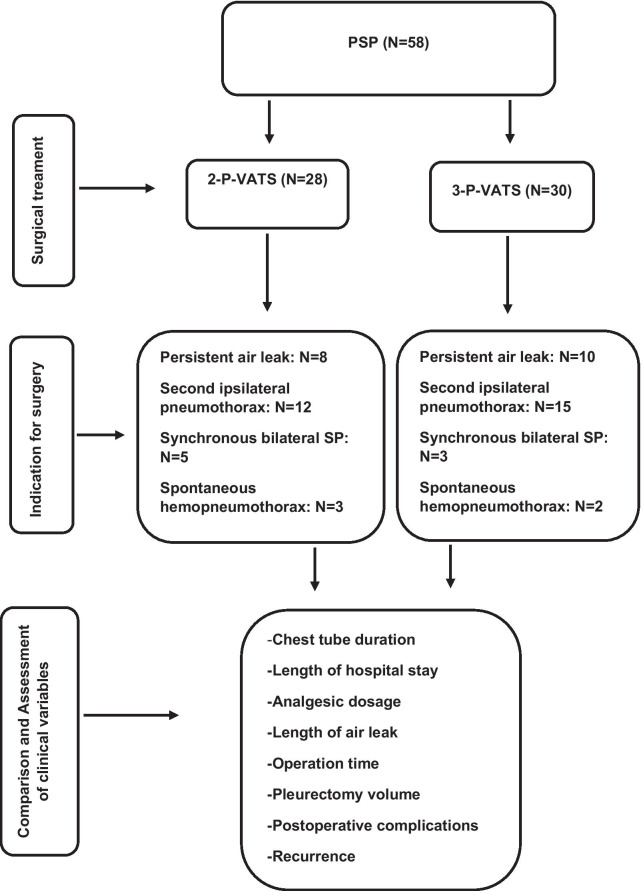
Flowchart displays methodological approach. *PSP* primary spontaneous pneumothorax, *2-P-VATS* Two-ports VATS, *3-P-VATS* Three-ports VATS

Figure 3 shows a summary of the methodological approach of this study.

### Statistical analysis

All data were analysed with the SPSS 25.0 software program (Statistical Package for Social Sciences; SPSS Inc., Chicago, IL, USA). Categorical variables were expressed as percentages and continuous variables were presented as mean. The means of categorical variables were compared by chi-square test or Fisher’s exact test, and continuous variables were compared by Student’s *t*-test. Statistical significance was considered at *p* < *0.05.*

## Results

Fifty-eight PSP patients were included in this study. The mean age of the patients was 25 years (range 18–48). Overall, there were 49 male and 9 female patients included. Thirty patients underwent 3-P-VATS, whereas 28 patients received 2-P-VATS. The mean age, sex, side of pneumothorax, smoking behaviour, BMI and pneumothorax size were similar in the 2-P-VATS group and the 3-P-VATS group. The clinical characteristics of the patients are summarised in Table 1.

Surgical characteristics of the two groups are listed in Table 2. There was no significant difference in the number of resected lung segments between both groups, suggesting a lack of selection bias based on the resected lung segments The mean operation time for the 2-Port-VATS was significantly shorter compared with the 3-P-VATS (65 min vs. 90 min, *p* = *0.017*). Furthermore, patients operated using 3-P-VATS required a significantly higher total dose of opioid and non-opioid analgesics per stay, compared with patients treated by 2- P-VATS (opioid: 30 mg vs. 15 mg; *p* = *0.021*; non-opioid: 16 g vs. 20 g; *p* = *0.010*). 2- P-VATS patients had a significantly shorter LOS compared with 3-P-VATS patients (7 days vs. 9 days; *p* = *0.012*). The duration of chest tube was longer in the 3-P-VATS group compared with the 2-P-VATS group, although this difference did not reach statistical significance (5.5 days vs. 7 days; *p* = *0.228*). Three patients in the 3-P VATS group suffered a postoperative haemothorax, while none of the patients in the 2-P VATS group was affected. Although we observed no significant difference, the pleurectomy volume was larger in the 3-P-VATS patients compared to the patients who underwent 2-P-VATS. One of the patients with postoperative hemothorax required recurrent VATS, the other two patients were successfully treated conservatively. All patients with prolonged air leak received conservative treatment until full recovery. During a median follow-up period of 61.6 months, 5 (16.7%) patients in the 3-P-VATS group suffered a recurrence, whereas only 2 (7.1%) patients in the 2-P-VATS group experienced recurrence. However, this difference did not reach statistical significance (*p* = *0.274*).

## Discussion

In the last decades, thoracic surgery has evolved from thoracotomies to video assisted thoracoscopic surgery (VATS) as the gold standard. While three port VATS still remains the standard-of-care in most centres due to the accessibility, recent technical developments are leading to a reduction in access ports. While this may improve postoperative performance such as reduced paraesthesia, analgesic use and LOS [6–11], the more limited access may reduce the operative results. While single-port VATS has been heralded as new minimal access VATS in selected indications and specialized centres, two- and three-port VATS remains the gold standard in most settings. Yet, comparisons of 3-P-VATS with 2-P-VATS have been rarely reported and the impact on postoperative performance as well as effectiveness of the surgical therapy remain elusive.

In the study of Lin F et al. [12] with 23 PSP patients who underwent 2-P-VATS and 73 patients with 3-P-VATS, mean operation time, average LOS and average postoperative chest tube duration were not significantly different between both groups. Similar to our results, postoperative pain was significantly lower in the 2-P-VATS group compared with the 3-P-VATS group. In another recent study of Kutluk AC et al. [8] including 45 patients operated by 2-P-VATS und 45 patients by 3-P-VATS, no significant difference was observed in mean operation time, LOS, duration of chest tube drainage, recurrence rates and postoperative pain.

In our study, 28 patients underwent 2-P-VATS and 30 patients received 3-P-VATS. In contrast to the above studies [8, 12], we observed a significant difference in operation time, LOS and postoperative dose of analgesic between both groups. Patients operated by 2-P-VATS had a significantly reduced LOS, less postoperative pain and shorter operation time compared with patients operated by 3-P-VATS. In terms of postoperative complications, we observed a significantly higher rate of hemothorax in the 3-P-VATS group. Although not reaching statistical significance, the larger volume of resected parietal pleura in the 3-P-VATS groups (14.3 vs. 12.8 cm^3^), as well as the additional port access, may have contributed to the higher rate of hemothorax in the 3-P-VATS group. As described in previous studies, VATS with additional pleurectomy is associated with reduced recurrence rates [14–16]. However, the volume of pleurectomy seems to affect postoperative outcome in terms of complications and postoperative pain. Regarding our study groups, patients who underwent 3-P-VATS suffered a high complications rate and had a high analgesic use compared to patients after 2-P-VATS. We attributed these results to the high pleurectomy volume and the additional port access implemented during 3-P-VATS.

To assess the rate of recurrence, all patients were followed-up for a mean period of 61.6 months. During this period, 5 (16.7%) and 2 (7.1%) patients from the 3-P and 2-P VATS group, respectively, suffered a recurrence. Despite the lower volume of resected pleura in the 2-P-VATS group, there was no significant difference in terms of recurrence rates between both groups.

In previous studies [6–12], postoperative pain was assessed using the visual analogue scale (VAS), which is a subjective measure for pain. In our study, we used the cumulative postoperative dosage of opioid and non-opioid analgesics per patient as an objective surrogate for postoperative pain. Unlike the VAS score, which may give a one-time measurement including the patient’s emotional and psychological state, the quantification of applied analgesics allows to assess average pain levels over a longer period. We found a significantly reduced opioid and non-opioid dosage in the 2-P-VATS group, most likely due to the reduced port access. To our knowledge, our study is the first study that elucidates pain-related analgesic use after VATS for patients with PSP.

Our results demonstrate that 2-P-VATS in the treatment of PSP leads to a better postoperative outcome and earlier recovery compared with the conventional 3-P-VATS.

As a retrospect analysis of a limited cohort, this study carries the limitations inherent to this approach. Due to the study period, not all operations were performed by the same surgeon. Nevertheless, our findings, especially the significantly higher rate of postoperative hemothorax observed in our cohort in the 3-P-VATS group, should be further elucidated in larger, randomized controlled trials.

## Conclusion

Our data demonstrate that 2-P-VATS is safer and as effective as 3-P-VATS in the treatment of PSP. It is associated with decreased postoperative pain, reduced length of hospital stay and fewer postoperative complications, indicating that 2-P-VATS should be considered standard-of-care in the treatment of PSP.

## Data Availability

The datasets used and/or analysed during the current study are available from the corresponding author on reasonable request.

## Abbreviations

PSP: Primary spontaneous pneumothorax
VAS: Visual analog scale
VATS: Video-assisted thoracoscopic surgery
3-P-VATS: Three-port VATS
2-P-VATS: Two-port VATS
LOS: Length of hospital stay
PV: Pleurectomy volume
BMI: Body mass index
